# An MR-based brain template and atlas for optical projection tomography and light sheet fluorescence microscopy in neuroscience

**DOI:** 10.3389/fnins.2024.1328815

**Published:** 2024-03-27

**Authors:** Stefanie M. A. Willekens, Federico Morini, Tomas Mediavilla, Emma Nilsson, Greger Orädd, Max Hahn, Nunya Chotiwan, Montse Visa, Per-Olof Berggren, Erwin Ilegems, Anna K. Överby, Ulf Ahlgren, Daniel Marcellino

**Affiliations:** ^1^Department of Clinical Microbiology, Umeå University, Umeå, Sweden; ^2^The Laboratory for Molecular Infection Medicine Sweden (MIMS), Umeå University, Umeå, Sweden; ^3^Department of Medical and Translational Biology, Umeå University, Umeå, Sweden; ^4^The Rolf Luft Research Centre for Diabetes and Endocrinology, Karolinska Institutet, Stockholm, Sweden

**Keywords:** mesoscopic imaging, OPT, LSFM, MRI, neuroimaging, brain template

## Abstract

**Introduction:**

Optical Projection Tomography (OPT) and light sheet fluorescence microscopy (LSFM) are high resolution optical imaging techniques, ideally suited for ex vivo 3D whole mouse brain imaging. Although they exhibit high specificity for their targets, the anatomical detail provided by tissue autofluorescence remains limited.

**Methods:**

T1-weighted images were acquired from 19 BABB or DBE cleared brains to create an MR template using serial longitudinal registration. Afterwards, fluorescent OPT and LSFM images were coregistered/normalized to the MR template to create fusion images.

**Results:**

Volumetric calculations revealed a significant difference between BABB and DBE cleared brains, leading to develop two optimized templates, with associated tissue priors and brain atlas, for BABB (OCUM) and DBE (iOCUM). By creating fusion images, we identified virus infected brain regions, mapped dopamine transporter and translocator protein expression, and traced innervation from the eye along the optic tract to the thalamus and superior colliculus using cholera toxin B. Fusion images allowed for precise anatomical identification of fluorescent signal in the detailed anatomical context provided by MR.

**Discussion:**

The possibility to anatomically map fluorescent signals on magnetic resonance (MR) images, widely used in clinical and preclinical neuroscience, would greatly benefit applications of optical imaging of mouse brain. These specific MR templates for cleared brains enable a broad range of neuroscientific applications integrating 3D optical brain imaging.

## 1 Introduction

Three-dimensional (3D) visualization of specific cell populations, protein expression patterns or pathologic markers at the whole brain level represents an invaluable tool in neuroscience. Optical projection tomography (OPT) and light sheet fluorescence microscopy (LSFM) are high-resolution optical 3D imaging techniques, enabling visualization of specifically labeled targets in mesoscopic sized (mm-cm range) transparent specimen (Sharpe et al., 2002; Dodt et al., 2007). Therefore, these optical techniques harbor great suitability for *ex vivo* whole rodent brain imaging, providing information at cellular resolution in the intact brain (Alanentalo et al., 2007; Hansen et al., 2020). In line with other functional imaging modalities, OPT and LSFM display high sensitivity and specificity for their target, but offer only very limited anatomical information. Considering the highly compartmentalized anatomy of the brain and the specific roles these regions fulfill, it is of the utmost importance to be able to map fluorescent signals, acquired by OPT or LSFM, to annotated brain regions. The possibility to anatomically map protein expression profiles and perform 3D quantification and statistics on these images, would greatly benefit the application of optical mesoscopic imaging in neuroscience.

The Common Coordinate Framework version 3 (CCFv3) of the Allen Institute of Brain Sciences (AIBS) (Sunkin et al., 2013; Oh et al., 2014; Wang et al., 2020) has been used for co-registration and quantitative analyses of 3D optical brain images (Renier et al., 2014; Furth et al., 2018) and has formed the basis for LSFM-specific brain templates based on optically cleared brains, which were successfully applied to study drug effects in the whole brain (Salinas et al., 2018; Perens et al., 2021). The CCFv3 template, based on serial two-photon tomography (STPT) imaging (Wang et al., 2020), has a 10 μm^3^ isotropic voxel size and contains 658 annotated individual structures. Its applicability has been demonstrated as well suited to link connectivity patterns, functional properties and cellular architecture (Oh et al., 2014; Goubran et al., 2019; Qu et al., 2022). Nevertheless, the CCFv3 reference brain is about 15% larger than the average of the individual brains included in the template (Wang et al., 2020). As the deformations induced by tissue clearing, required for 3D optical imaging often include shrinkage, a larger brain template is sub-optimal for the creation of fusion images with 3D optical whole brain images. Furthermore, STPT and OPT/LSFM can induce additional differential deformations and provide distinct tissue contrast, which can both hamper co-registration and normalization to the template. The LSFM-based templates, on the other hand, are based on tissue autofluorescence and therefore may provide suboptimal tissue contrast and anatomical detail to distinguish all brain regions.

Magnetic Resonance Imaging (MRI) is well known to provide detailed anatomical brain images due to its ability to acquire images with high resolution and exquisite tissue contrast. This makes MR images ideally suited as anatomical reference for the creation of fusion images, as exemplified by the increased interest in PET-MR for brain imaging. The most straightforward way to create fluorescence-MR fusion images is by using an MRI-based mouse brain template, of which several are currently available (Dorr et al., 2008; Hikishima et al., 2017; Barriere et al., 2021). However, these templates originate from MR images acquired either *in vivo* or *ex vivo in situ* (in the skull) while OPT and LSFM are acquired after brain removal from the skull and following extensive processing and tissue clearing which are known to exert effects on brain size and morphology (Wan et al., 2018). For OPT and LSFM, it would therefore be ideal to create fusion images with an MR-based brain template, containing individual MR images acquired after tissue preprocessing and clearing, to correct for all brain deformations induced by these processes.

Here, we present the creation of the novel MR-based, high resolution (40 μm^3^ isotropic voxel size) Optically Cleared UMeå brain template (OCUM), designed from *ex vivo* T1-weighted MR images, acquired after tissue preprocessing and clearing for optical imaging, with its associated tissue priors and corresponding atlas annotating 336 volumes of interest (VOIs). Two versions of the template and atlas are presented, each optimized for two distinct clearing method that are most often used for 3D optical brain imaging and that have differential effects on brain size. Furthermore, we provide a workflow to work with 3D optical datasets (up to 10 GB/ channel), usually saved as large collections of TIFF-files, using the Neuroimaging informatics technology initiative (NifTI) file format. Thereby, all transformations on optical brain images can be executed using Statistical Parametric Mapping (SPM), identical as for PET and MR images. Finally, we demonstrate the utility of OCUM by creating fluorescence-template fusion images with 3D optical images mapping dopamine transporter (DAT) expression, viral brain infection, the 18 kDa translocator protein (TSPO), and the optic nerve innervation of lateral geniculate nucleus of the thalamus and superior colliculus. In all cases, fusion images allowed precise anatomical identification of brain regions with fluorescent signal. As such, these templates will significantly benefit the applicability of mesoscopic optical imaging in neuroscience.

## 2 Materials and methods

### 2.1 Ethics declaration

All animal experiments were approved and performed according to the guidelines of the regional Animal Research Ethics Committee of Northern Norrland, the Animal Review Board at the Court of Appeal of Northern Stockholm and by the Swedish Board of Agriculture (Ethical permits: A35-2016, A9-2018 and A41-2019). Reporting regarding all *in vivo* experiments was performed compliant with the ARRIVE guidelines.

### 2.2 Animals

Eight- to eleven-week-old male C57Bl/6 J mice (*n* = 10) were purchased from Jackson Laboratories (Bar Harbor, ME, United States) or Charles River (Wilmington, MA, United States). Interferon alpha/beta receptor knockout (IFNAR^−/−^) (*n* = 6, 4 M/2F) (kindly provided by N.O Gekara, Umeå University) (Muller et al., 1994), interferon-beta promoter stimulator-1 knockout (IPS-1^−/−^) (*n* = 3, 1 M/2F) and Viperin^−/−^ (*n* = 1, F) mice (a kind gift from Peter Cresswell, Department of Immunobiology, Yale University School of Medicine) were bred at the Umeå Centre for Comparative Biology (UCCB) at Umeå Univeristy. Animal experiments were conducted at UCCB and at the department of Molecular Medicine and Surgery at Karolinska Institutet (MMK). Following euthanasia using O_2_ deprivation or anesthesia using 60 mg/mL pentobarbital (APL, Kungens Kurva, Sweden), all animals (*n* = 19) were transcardially perfused using 20 mL PBS followed by 20 mL 4% paraformaldehyde (PFA) in PBS whereafter brains were harvested for *ex vivo* analyses.

### 2.3 Viral infection

After sedation with ketamine (100 μg/g body weight) and xylazine (5 μg/g body weight) or isoflurane, animals were intracranially inoculated with 100 focus forming units (FFU) Langat virus (LGTV) strain P21or with PBS (Mock). Mice were euthanized at humane endpoint, i.e., when they developed one category 4 symptom such as: >20% weight loss, bilateral eye infection, diarrhea, or hind-limb paralysis; or when they developed 3 category 3 symptoms such as: >15% weight loss, unilateral eye infection, facial edema, ruffled fur or overt bradykinesia and/or development of stereotypes.

### 2.4 Cholera toxin B tracing

Animals were anesthetized using isoflurane and injected with 4 μL of 0.1% (w/v) cholera toxin subunit B conjugated to AlexaFluor-647 (CTB, Thermofisher Scientific) diluted in PBS directly into the anterior chamber of the eye (ACE) using a Hamilton syringe (702 SN SYR, Tillquist). The needle was left inside of the eye for 2–6 min to avoid any CTB leakage. The contralateral eye was injected with an equivalent volume of PBS as a control for autofluorescence. To ensure sufficient time for anterograde tracing form the eye to the brain, mice were euthanized 7 days post CTB injection. Afterwards, mice we transcardially perfused using 25 mL saline containing 0.5% (v/v) heparin followed by 25 mL 4% PFA in 0.4 M Sörensen’s buffer (pH 7.35) and 25 mL ice-cold PFA. Finally, brains with the intact optical nerves and eyes attached were dissected from the skull post-fixed in 4% PFA for 4 h.

### 2.5 Whole mount immunohistochemistry and optical clearing

PFA-fixed brains were fluorescently immunolabeled and processed for OPT as described previously (Alanentalo et al., 2007; Eriksson et al., 2013). Briefly, brains were dehydrated using stepwise gradients of methanol (MeOH), permeabilized by 4 cycles of repetitive freeze-thawing in MeOH at −80°C and bleached overnight (ON) in MeOH:H_2_O_2_:DMSO (2:3:1) at room temperature (RT) to quench tissue autofluorescence. For immunolabeling, brains were rehydrated into TBST (50 mM Tris–HCl pH7.4, 150 mM NaCl and 0.1% TritonX-100) and labeled with primary (recombinant rabbit anti-TSPO (1:1000) (ab109497, Abcam, Camebridge, United Kingdom), rabbit anti-DAT (1:400) (clone 1D2 ZooMAb, n°: ZRB1525, Sigma Aldrich, St. Louis, MO, United States) or chicken anti-viral non-structural protein (NS5) (1:1000) (Agrisera AB, Vännäs, Sweden) (produced according to the manufacturer’s protocol using the peptide sequence of NS5 from TBEV strain Torö, GenBank: DQ401140)) and secondary (goat anti-rabbit Alexa-594 (1:500) (A-11037, Thermo Fisher, Scientific, Waltham, MA, United States), donkey anti-rabbit Alexa-594 (1:500) (ab150064, Abcam) or goat-anti chicken Alexa-680 (1:500) (ab175779, Abcam)). For some brains, DAPI (1:2000) was added to the secondary antibody dilution. After immunolabeling, all brains were mounted in 1.5% low melting point agarose (SeaPlaque, Lonza, Basel, Switzerland) and optically cleared using benzyl alcohol: benzyl benzoate (1:2) (BABB) or dibenzyl ether (DBE) (Sigma-Aldrich).

### 2.6 Optical projection tomography

OPT image acquisition was performed on an in-house developed near-infrared OPT (NiR-OPT) system, as described by Eriksson et al. (2013). A zoom factor of 1.25 (cholera toxin) or 1.6 (all other brains) was applied, which resulted in a respective isotropic voxel dimension of 21 μm^3^ or 16.5 μm^3^. For cholera toxin, OPT images were acquired using the following settings: Ex: 630/50 nm, Em: 665/95 nm (exposure time: 3000 ms). For virus infected brains, OPT images were acquired using: Ex: 665/45 nm, Em: 725/50 nm (exposure time: 7000 ms). For all other targets, OPT images were acquired using Ex: 580/25 nm, Em: 625/30 nm (exposure time: 500 ms) (TSPO) or 3,000 ms (DAT). All tissue autofluorescence images were acquired with the same settings namely: Ex: 425/60 nm, Em 480LP nm (exposure time: 200 ms). To increase the signal-to-noise ratio (SNR) of the labeled molecules in the brains, the pixel intensity range of all images were adjusted to display minima and maxima and a contrast limited adaptive histogram equalization (CLAHE) algorithm with a tile size of 16 × 16 was applied to the projection images acquired in the fluorescent signal channels. Tomographic reconstruction with additional misalignment compensation and ring artifact reduction was performed using NRecon software v.1.7.0.4 (Skyscan microCT, Bruker, Belgium). Afterwards, OPT images displaying both the targeted signals and the tissue autofluorescence signals, were reconstructed into DICOM format using NRecon software, followed by their conversion into NifTi format using the PMOD view tool (version 4.2, PMOD Technologies Inc., Zurich, Switzerland).

### 2.7 Light sheet fluorescence microscopy

The brain stained for TSPO and imaged by NiR-OPT was consequently rescanned using an UltraMicroscope II (Miltenyi Biotec, Germany) including a 1x Olympus objective (Olympus PLAPO 2XC) coupled to an Olympus MVX10 zoom body, providing 0.63x up to 6.3x magnification with a lens corrected dipping cap MVPLAPO 2x DC DBE objective (Olympus). The UltraMicroscope II is equipped with the white laser SuperK EXTREME (NKT photonics Birkerød, Denmark). The cleared brain was immerged in BABB and magnification was set to 0.63x. For image acquisition, left and right light sheets were blend merged with a 0.14 numerical aperture, resulting in a light sheet z-thickness of 3.87 μm and 80% width, while using a 12-step contrast adaptive dynamic focus across the field of view. LSFM images were acquired with the following settings: Ex: 250/25, Em: 625/30 (for AlexaFluor-594 channel) and Ex: 470/40, Em: 525/50 (for the autofluorescence channel). Image sections with a step size of 10 μm were generated by Imspector Pro software (v7.1.15, Miltenyi Biotec Gmbh, Germany) and stitched together using the implemented TeraStitcher script (v.9). The obtained images were then converted into NifTi files using Amira Avizo software (version 6.3.0, Thermo Fisher Scientific, Waltham, MA, United States) and resampled prior to co-registration.

### 2.8 MRI acquisition

After optical clearing with BABB (*n* = 10) or DBE (*n* = 9) and OPT scanning of selected brains, all brains (*n* = 19), were rehydrated into TBST, incubated in 0.29 M sucrose to remove the surrounding agarose and washed in PBS prior to MRI. T1-weightd images were acquired using a Modified Driven Equilibrium Fourier Transform (MDEFT) sequence with five repetitions (TR: 3000 ms; TE: 3 ms; TI: 950 ms; voxel size 40 × 40 × 40 μm^3^) on a 9.4 Tesla (T) preclinical MR system (Bruker BioSpec 94/20, Bruker Ettlingen, Germany), equipped with a cryogenic Radio Frequency (RF) coil (MRI CryoProbe, Bruker) running Paravision 7.0 software. Data were exported in DICOM format using Paravision routines followed by image conversion from DICOM to NifTI format using the dcm2nii tool in MRIcron. The individual repetitions of each scan were realigned and averaged using statistical parametric mapping (SPM8) (the Wellcone Trust Centre for Neuroimaging, UCL, London, U.K.) implemented in Matlab (R2014a, The MathWorks Inc., Natick, MA, United States).

### 2.9 OCUM template and atlas creation

Initially, two distinct templates: namely one specifically for BABB (*n* = 10) and DBE (*n* = 9) cleared brains, were created using bias corrected (SPM8) MR images, which were realigned and averaged using serial longitudinal registration (SLR) in SPM12, implemented in Matlab (R2015b, The MathWorks Inc.). Then, all individual MR images were co-registered (separation: 0.08 and 0.04 mm, 4th degree B-spline interpolation) to their respective template. Co-registration was followed by normalization (no affine regularization, trilinear interpolation, 0.72 mm smoothing) of all individual BABB brains to the DBE template and vice versa. Consequently, the final OCUM and iOCUM templates were created by rerunning SLR on all brains (*n* = 19) both in BABB and DBE size, respectively. Both for OCUM and iOCUM, specific segments and tissue probability maps (TPMs) were created using a 2-step segmentation and diffeomorphic anatomical registration through exponentiated lie algebra (DARTEL) (Ashburner, 2007) pipeline, initially based on in-house generated tissue priors (Mediavilla et al., 2022). Briefly, a primary segmentation and DARTEL algorithm was applied to the individual MR images of both templates to generate preliminary tissue priors for both OCUM and iOCUM, using the toolbox SPMmouse (Sawiak et al., 2014). Thereafter, the complete process (segmentation + DARTEL) was repeated using the tissue priors generated from the previous step to produce accurate template specific TPMs for both templates. To create OCUM and iOCUM atlas labels, the DSURQE atlas labels were normalized (no affine regularization, trilinear interpolation) to both OCUM and iOCUM space. Anatomical accuracy of all the volumes of interest (VOIs) were visually assessed by two independent readers with profound knowledge of murine brain anatomy and, when required, manually adapted using the PMOD VOI tool.

### 2.10 Creation of fusion images with OCUM template

Initially, both the autofluorescence image and the OPT image displaying specific signals were reoriented manually in SPM8 to the templates orientation and their origins were set tangent to the upper edge of the brain at Bregma. For co-registration of OPT and MR images, voxel-to-voxel affine transformation matrices were calculated using the autofluorescence OPT images and applied to those displaying the specifically targeted signals. To further improve the fusion images and enable image warping, binary masks of the autofluorescence OPT images were created in ITK-SNAP version 3.8.0 (Yushkevich et al., 2006) which were consequently normalized to a binary OCUM template mask. Both co-registration and normalization were performed in SPM8, using the SPMmouse toolbox. Finally, fusion images were created in the PMOD view tool and 3D images were created in Amira-Avizo software (version 6.3.0, Thermo Fisher Scientific).

## 3 Results

### 3.1 Inadequate anatomical referencing with autofluorescence

**Figure 1:**
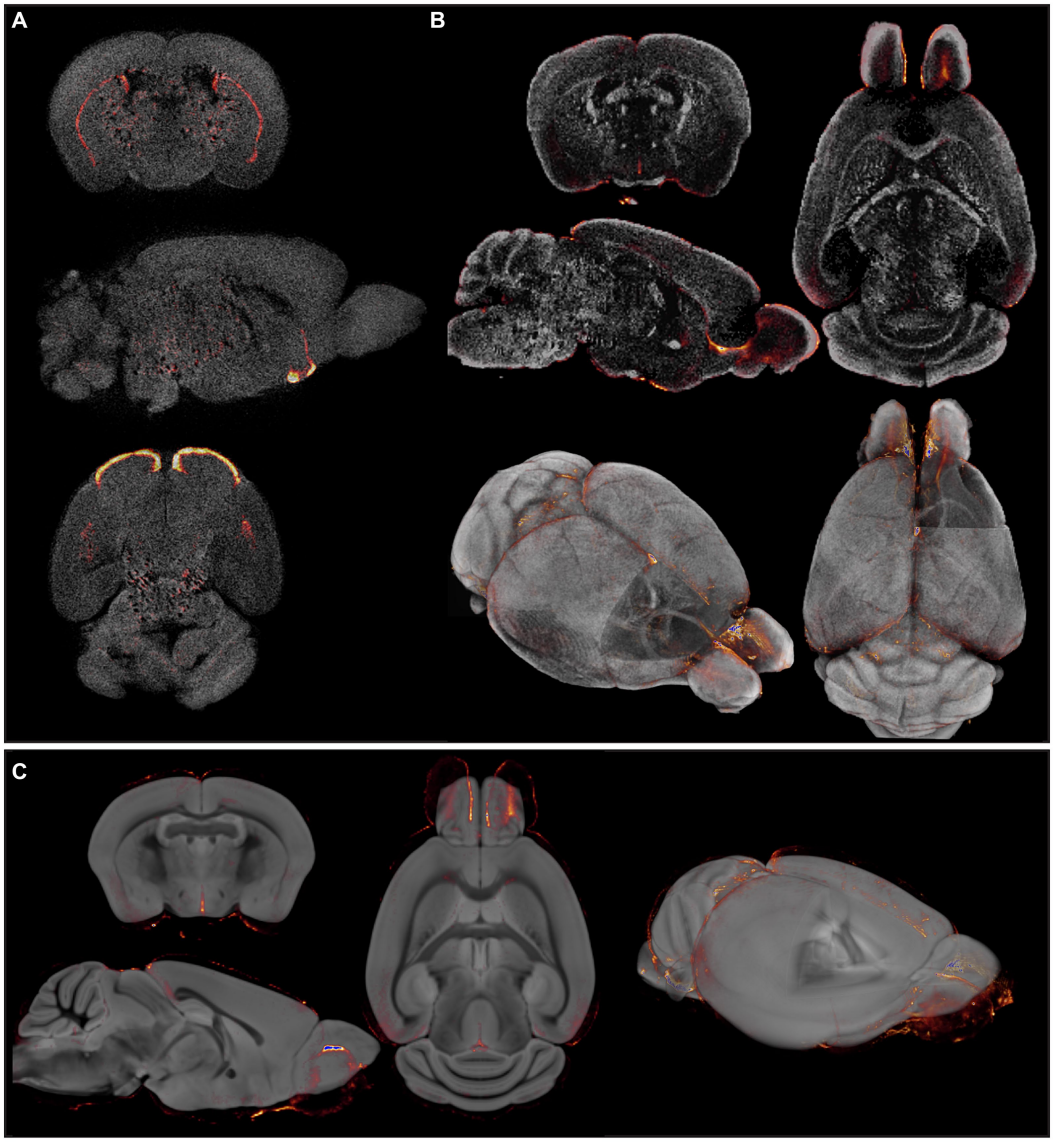
Insufficient and/or false anatomical mapping with existing methods. **(A)** Overlay of DAT signal acquired by OPT with the anatomy, reconstructed based on tissue autofluorescence. **(B)** Overlay of viral signal acquired by OPT with the anatomy, acquired based on DAPI staining. **(C)** Co-registration of viral signal acquired by OPT with the TMBTA results in anatomical misalignment and differences in brain size.

It is well known that brain tissue generally displays a high level of autofluorescence. Nevertheless, as shown in Figure 1A, it is nearly impossible to identify specific anatomical brain structures displaying dopamine transporter signal on native OPT-acquired autofluorescence images. Therefore, we first aimed to improve the autofluorescence anatomical signal by combining it with DAPI staining and optimize the reconstruction parameters for better contrast and more anatomical details and, overlayed the optical signal, in this case representing viral infection, onto an improved anatomical image (Figure 1B). Although tissue contrast between grey matter (GM) and white matter (WM) regions was increased, as well as an improved delineation of deep nuclei, anatomical detail remained insufficient to identify the majority of anatomical regions, mainly in cortical areas. In theory, contrast could be further improved by enhancing autofluorescence, for example by omitting the bleaching step during tissue pre-processing. However, to obtain high signal-to-noise ratios for antibody-targeted optical signals, autofluorescence should remain as low as possible. Therefore, accurate anatomical mapping of 3D optical brain signals cannot be obtained from tissue autofluorescence alone.

Since several MR-based brain templates with their corresponding atlases are available, we then aimed to create fluorescence-MR fusion images using the Turone Mouse Brain Template and Atlas (TMBTA) (Barriere et al., 2021), a recent *ex vivo* brain template acquired *in situ* with 1,320 annotated regions of interest (ROIs). As depicted in Figure 1C, co-registration (voxel-to-voxel affine registration followed by 4th degree B-spline interpolation) of viral signal, acquired by OPT, and the TMBTA resulted in clear misalignment, mainly in olfactory bulb. This observation might be explained by differences in acquisition, namely *ex vivo in situ* for TMBTA vs. *ex vivo* for OPT, where the brain is removed from the skull and the olfactory bulbs do not remain in their original anatomical position. Apart from misalignment, a difference in brain size between the template and the OPT signal can be observed from Figure 1C, which is likely caused by differences in image acquisition (*in situ* vs. *ex vivo*) in combination with the harsh chemical treatments required for optical clearing (Wan et al., 2018). Neither mismatch nor size differences after co-registration could be resolved by consequent normalization. Taken together, accurate anatomical mapping of 3D optical brain signals could not be obtained from tissue autofluorescence, nor from existing MR-based mouse brain templates.

### 3.2 Differential effects of clearing agents on brain volume

**Figure 2:**
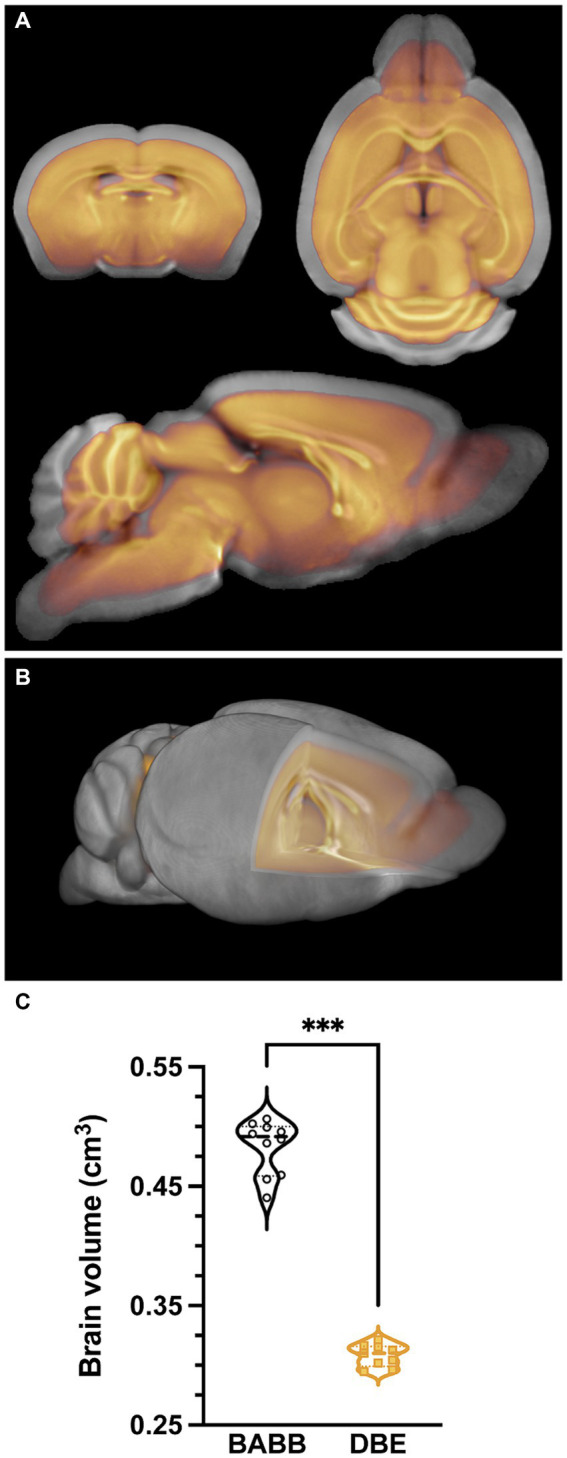
Differential effects of clearing methods on brain volume. **(A)** overlay of the average BABB template (*n* = 10) (grey) and DBE template (*n* = 9) (orange), indicating a clear difference in brain size. **(B)** 3D overlay of the average BABB (grey) and DBE (orange) templates, wherein the DBE template lies completely within the average BABB brain. **(C)** Brain volume comparison of the average BABB and DBE brain, respectively. Values are expressed in cm^3^. The average brain volume was significantly lower (***p < 0.001) for DBE cleared brains (0.308 ± 0.009 cm^3^) as compared to BABB cleared brains (0.483 ± 0.023 cm^3^).

To overcome the issues exemplified in Figure 1 and to obtain a representative anatomical reference for optically cleared brains that include discrete morphological changes (Wan et al., 2018) produced during pre-processing and brain clearing, we acquired *ex vivo* structural T1-weighted images of brains that were subjected to tissue processing and optical clearing with benzyl alcohol-benzyl benzoate (BABB)/Murray’s clear (*n* = 10) (Dodt et al., 2007; Becker et al., 2013) or with dibenzyl ether (DBE)/iDISCO (*n* = 9) (Renier et al., 2014), two frequently used protocols for optical tissue clearing, especially for brain. To investigate the effects of both BABB and DBE clearing on brain size and morphology, we initially created two individual brain templates, namely one for BABB cleared brains and one for DBE cleared brains, respectively. These individual templates were calculated as the mid-point average from a serial longitudinal registration of all individual, bias-corrected brains. Head-to-head comparison of the BABB and DBE templates revealed a clear difference in size, in which the BABB template was significantly larger than the DBE template (Figure 2). Furthermore, the size difference could not be attributed to sole differences in cortical shrinkage since a clear mismatch was observed in WM tracts and deep nuclei when superimposed in an identical image space (Figures 2A,B). Segment-based brain volume calculations on individual T1-weighted images revealed that DBE-cleared brains (0.308 cm^3^ ± 0.009) were significantly smaller (*p* < 0.001) when compared to BABB-cleared brains (0.483 ± 0.023 cm^3^) (Figure 2C). To further characterize the effects of tissue processing and clearing methods on the brain, we proceeded by comparing these volumes with brain volumes calculated from T1-weighted scans acquired *in vivo* (*n* = 62) and *ex vivo in situ* (*n* = 40), as well as with brain volumes based on autofluorescence of individual BABB and DBE brains (Supplementary Figure S1). We observed significant differences between *in vivo* brain volume (0.461 ± 0.011 cm^3^) and all other tested volumes. Both the *ex vivo* (0.437 ± 0.011 cm^3^) and DBE brains were significantly smaller (*p* < 0.001) than the *in vivo* volume, while BABB brain volume was significantly larger (*p* = 0.03). However, when BABB (0.343 ± 0.0517 cm^3^) and DBE (0.299 ± 0.071 cm^3^) brain volumes were calculated from autofluorescence, they were not significantly different (*p* = 0.12). Interestingly, BABB brain volume was significantly larger (*p* < 0.001) when calculated based on T1-weighted images (0.483 ± 0.023 cm^3^) as when calculated from OPT-based autofluorescence (0.343 ± 0.0517 cm^3^), which was not the case for DBE cleared brains (*p* = 0.94), indicating a distinct effect of BABB and DBE clearing on brain rehydration. Of note, no significant volume differences were observed between male and female mice when calculated from the T1-weighted images (*p* = 0.43), nor when calculated from autofluorescence (*p* = 0.11).

### 3.3 OCUM: T1-weighted reference template and atlas for optically cleared brains

**Figure 3:**
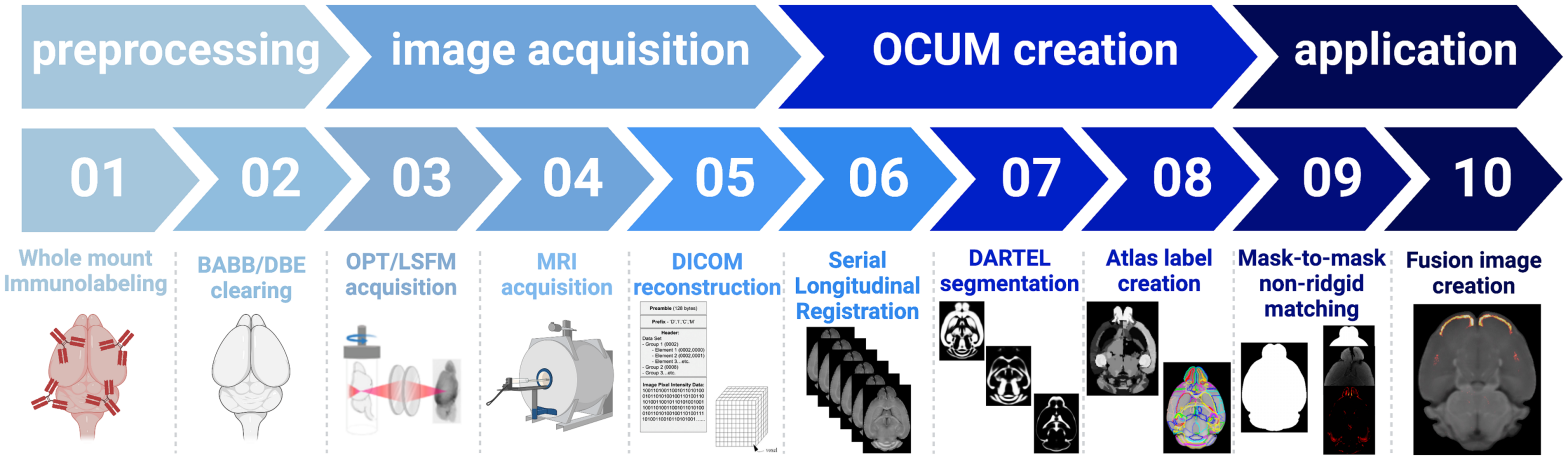
Schematic overview of the applied pipeline to obtain the OCUM and iOCUM brain templates with their associated tissue priors and atlas labels.

Due to the differential effects of BABB and DBE clearing and the significant size differences of the brain on T1-weighted images (Figure 2; Supplementary Figure S1), we decided on the creation of two brain templates containing all T1-weighted images (*n* = 19) namely the Optically Cleared UMeå (OCUM) brain template and atlas for BABB/Murray’s cleared brains and the iOCUM for DBE/iDISCO cleared brains, for which the DSURQE brain template and its atlas labels served as a foundation (Dorr et al., 2008; Richards et al., 2011; Ullmann et al., 2013; Steadman et al., 2014; Qiu et al., 2018). A schematic overview of the applied pipeline to create OCUM and iOCUM is provided in Figure 3. All image transformations were performed in SPM, using the SPMmouse toolbox (Sawiak et al., 2014). The outcome of all applied transformations was reviewed by two independent readers with caution for involuntary image flipping along the y-axis.

**Figure 4:**
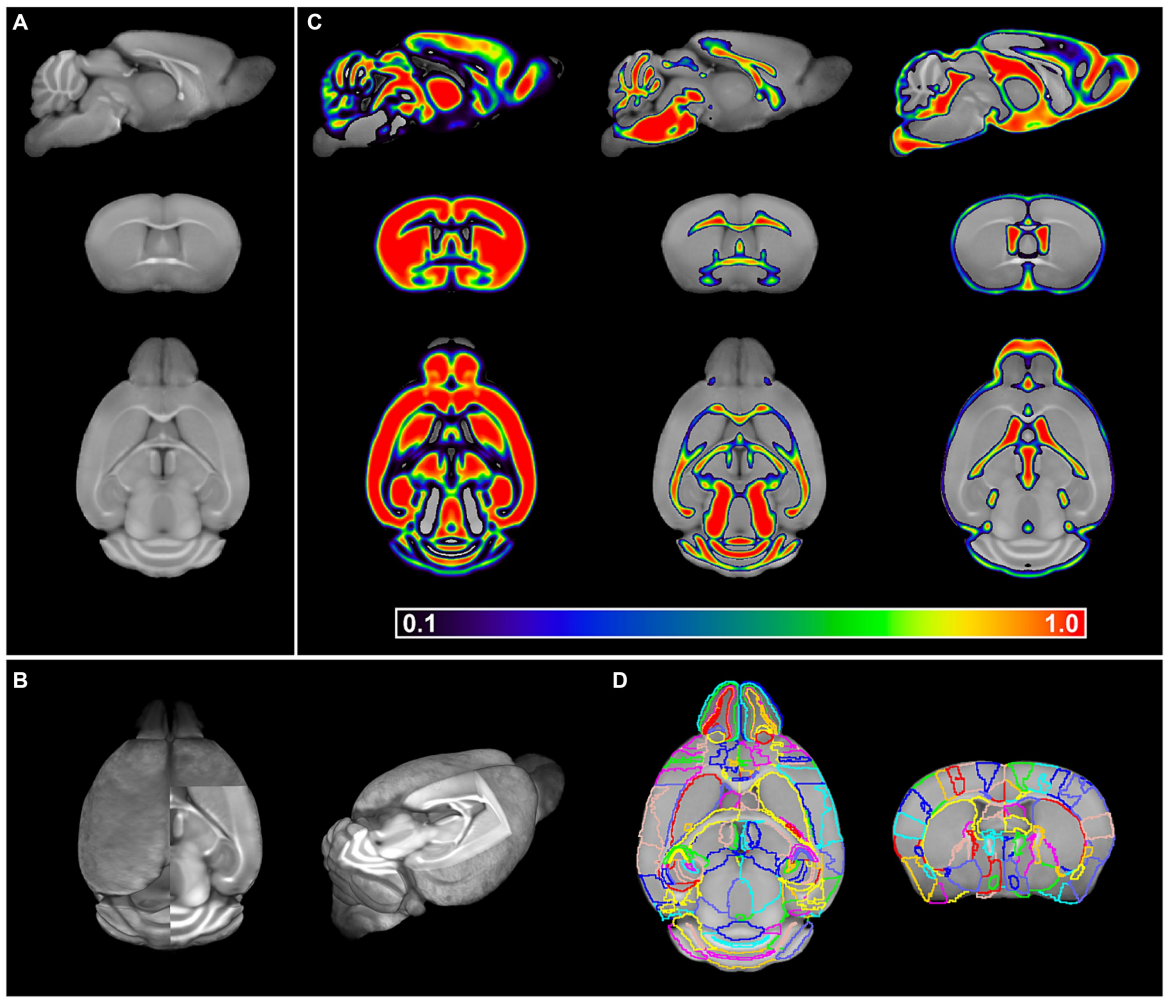
OCUM brain template and atlas. **(A)** sagittal, coronal and axial brain slice of the OCUM template (*n* = 19). **(B)** 3D representation of OCUM showing high GM and WM contrast in the template. **(C)** GM, WM and CSF probability maps associated with OCUM. **(D)** Volume of interest (VOI) delineation exemplified on a axial and coronal slice of OCUM.

In short, all individual brains were co-registered to their respective template whereafter individual BABB brains were normalized to the DBE template and vice versa. The calculation of the midpoint average of the serial longitudinal registrations of all individual MR images then resulted in two templates, OCUM for BABB and iOCUM for DBE size, respectively (Figures 4A,B). Then, we created tissue segments and tissue probability maps (TPM) for both templates. Initially, we used in-house tissue priors from *ex vivo in situ* acquired T1-weighted images to generate preliminary tissue priors for OCUM and iOCUM. Thereafter, we reran the algorithm to produce accurate and template specific TPMs for OCUM and iOCUM (Figure 4C), which we used for all following image transformations and analyses. Finally, we normalized the DSURQE atlas to OCUM and iOCUM space to delineate VOIs. Visual assessment by two independent readers showed that subcortical structures such as deep nuclei and anterior cortical regions were perfectly delineated by normalization while posterior cortical VOIs were manually adapted using the PMOD VOI tool for each template, resulting in OCUM and iOCUM specific atlas labels (Figure 4D; Supplementary Table S1).

Together, our final resources comprise of two high-resolution (40 μm^3^ isotropic voxel dimension) mouse brain templates, corresponding TPMs required for image transformations, a brain atlas with 336 annotated ROIs and a protocol to generate fluorescence-MR fusion images.

### 3.4 Accurate anatomical referencing of optical brain images

**Figure 5:**
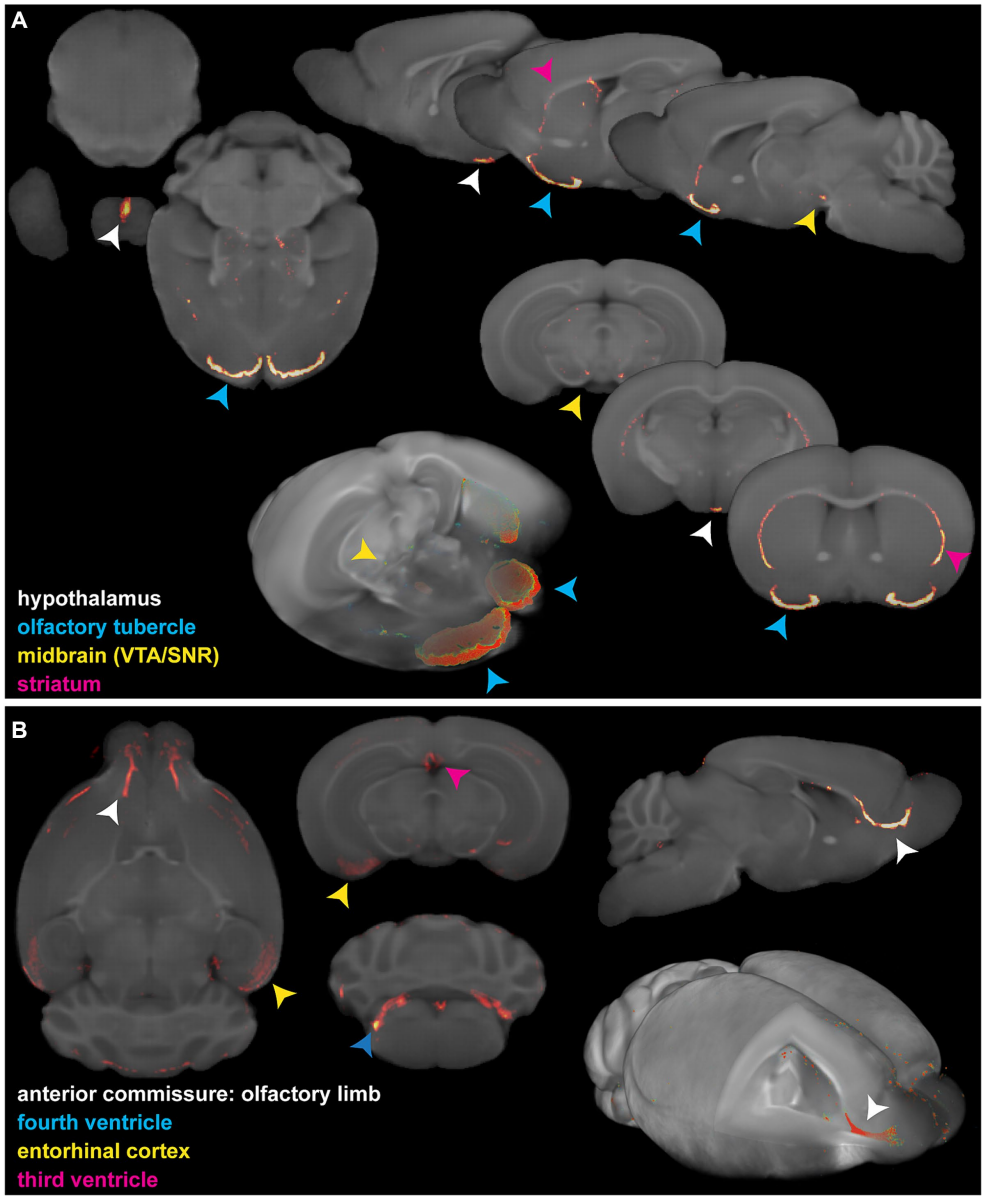
Fusion images of 3D optical DAT and viral signal with the OCUM template. **(A)** 2D and 3D representations of fusion images of DAT OPT signal in typical dopamine transporter expressing brain regions and OCUM. VTA = ventral tegmental area; SNR = Substantia Nigra. **(B)** 2D and 3D representations of fusion images of viral (NS5) OPT signal in infected brain regions and OCUM.

**Figure 6:**
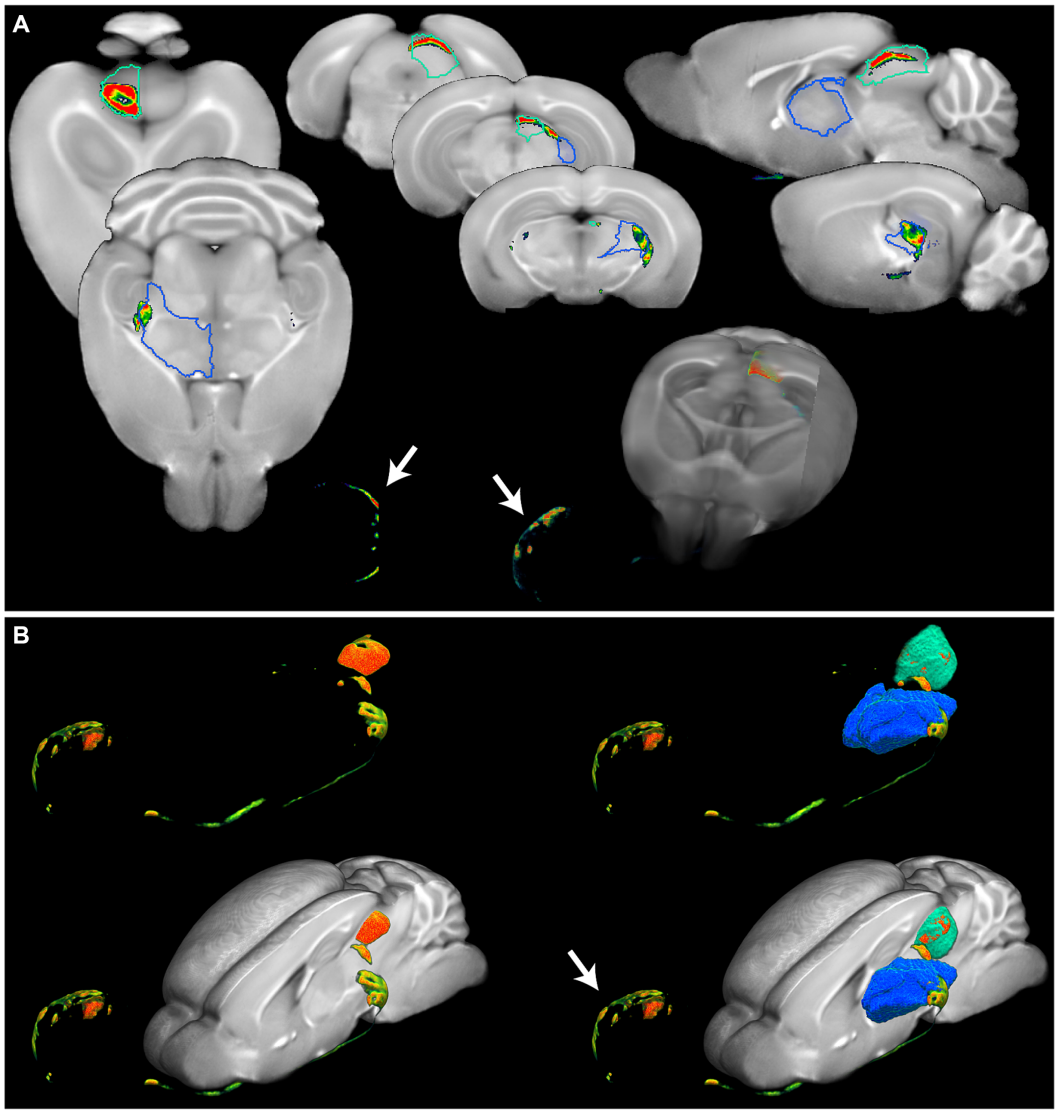
Detailed anatomical brain mapping of Cholera toxin B after intraocular injection. **(A)** Fusion images of OPT signal of cholera toxin B and OCUM displaying signal in the eye (white arrow) and distinct parts of the visual system. Optical signal can be observed in the dorsolateral geniculate nucleus of the thalamus (blue VOI) and the optic nerve layer of the superior colliculus (green VOI). **(B)** 3D representations of the optical cholera toxin B signal from the eye (white arrow) along the optical tract through the dorsolateral geniculate nucleus of the thalamus (blue VOI) to the optic nerve layer of the superior colliculus (green VOI), with and without the OCUM template.

**Figure 7:**
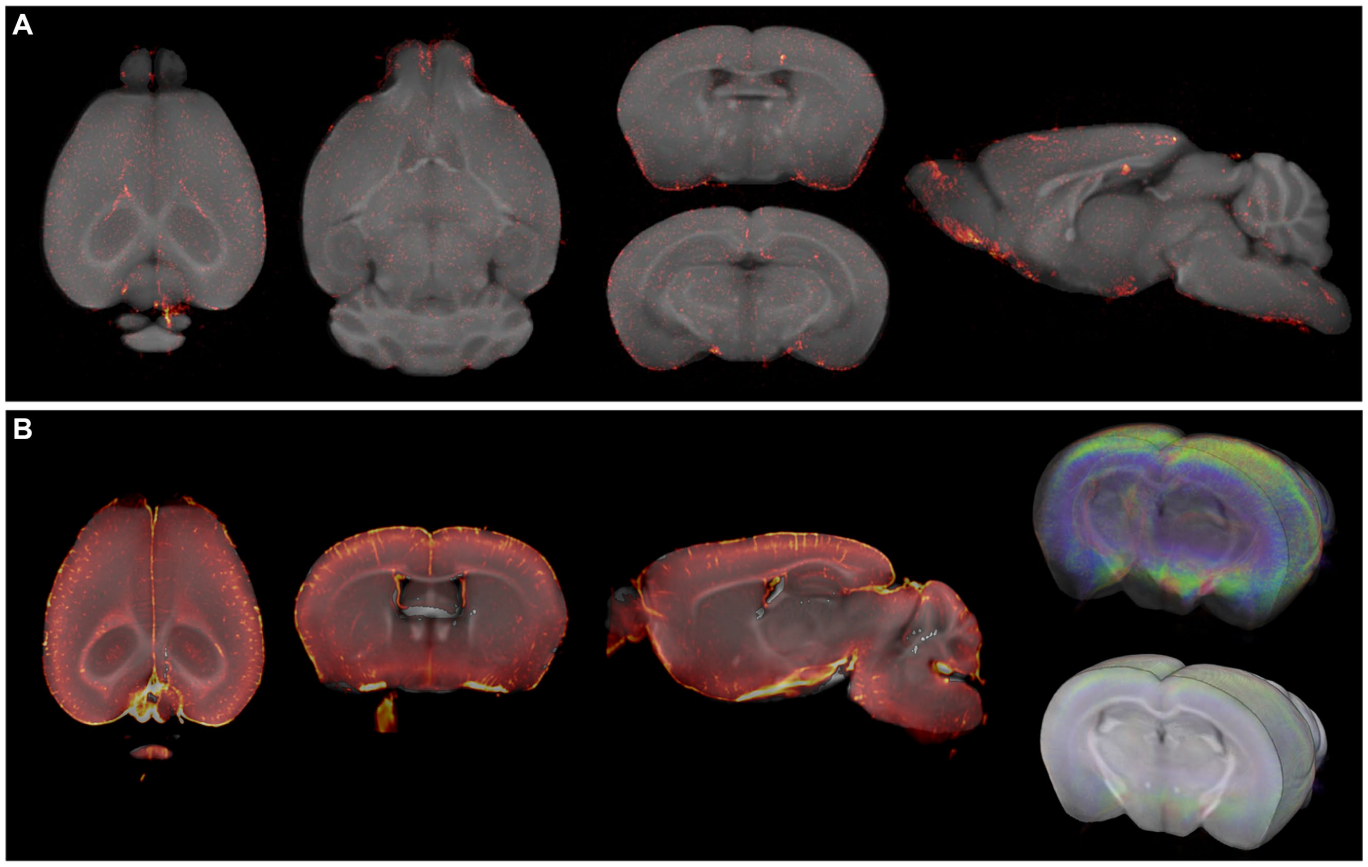
Fusion images of TSPO OPT and LSM signal with OCUM. **(A)** Fusion images of TSPO signal, targeting microglia, and OCUM. **(B)** Fusion images of TSPO LSFM signal and OCUM, showing high optical signal in cortical 4 due to microglial activation after viral infection, and intense vessel staining due to TSPO expression in endothelial cells.

To highlight the applicability of the newly designed templates, we created fusion images of 3D fluorescence signals, acquired from BABB and DBE cleared brains, with OCUM and iOCUM, respectively. Therefore, optical images were converted to NIFTI format (Figure 3) to allow image transformations in SPM. First, we co-registered the optical images to the applicable template. Voxel-to-voxel affine transformation matrices were calculated using autofluorescence and consequently applied to the image containing optical signal. To improve fusion image quality, normalization to the template is preferred to compensate for natural variation and tissue deformation due to dehydration and clearing. As shown in Figure 1 however, the autofluorescence images have limited anatomical detail, which hampers accurate normalization. Therefore, we created a mask of the autofluorescence image and normalized this mask to a similar (i)OCUM mask and applied identical transformations to the original images which resulted in near-perfect fusion images. For all tested cases, fusion images allowed for precise atlas-based anatomical identification of brain regions displaying fluorescent signal (Figures 5–7; Supplementary Figure S2). Figure 5 and Supplementary Figure S2 display fusion images from DAT and viral infection OPT with OCUM and iOCUM, respectively. For DAT (Figure 5A; Supplementary Figure S2A), we identified clear signal in the striatum, hypothalamus, olfactory tubercle, amygdala and even in the substantia nigra. All these regions are well-known to express DAT, which underlines the applicability of our templates and atlases. For viral infection (Figure 5B; Supplementary Figure S2B), fusion images allowed exact identification of viral (NS5) distribution and infected brain areas. For both BABB and DBE cleared brains, using OCUM and iOCUM, respectively, we observed viral signal in the third, fourth and lateral ventricles, olfactory limb of anterior commissure and entorhinal cortex, which is in line with previous observations (Chotiwan et al., 2023). We traced anterograde innervation following injection of fluorescently labeled cholera toxin B (CTB) in the anterior chamber of the eye (Figure 6). We observed clear localization of CTB signal within the contralateral LGN of thalamus and the superior colliculus. The match with brain regions of the visual system along the optical nerve, indicate anterograde innervation from the eye towards the visual cortex. Lastly, we created fusion images of both OPT (Figure 7A) and LSFM (Figure 7B) signal of TSPO, a well-known neuroinflammation marker. In contrast to the three previous examples, TSPO displays a diffuse, whole brain expression pattern rather than being expressed in distinct brain regions. Figure 7 clearly shows that the presented co-registration/normalization workflow also works for optical images originating from diffusely expressed markers. Both TSPO OPT (Figure 7A) and LSFM (Figure 7B) showed elevated signal in brain vasculature, which can be explained by TSPO expression in endothelial cells. Furthermore, next to detailed vessel staining, TSPO LSFM showed an increase in signal in cortical layer IV in virus infected brains (Figure 7B), which suggests higher expression levels of activated microglia in this highly myelinated cortical layer.

## 4 Discussion

Whole brain optical imaging is rapidly gaining interest and popularity to study protein expression profiles and disease markers in neuroscience. MR, on the other hand, is a non-invasive, well-established and common imaging modality used for both preclinical and clinical brain research. The use of these techniques, in combination with advanced image quantification, represents a powerful triad to discover insights into the healthy and/or diseased brain. Here we report, to our knowledge, the first T1-weighted MR-based high-resolution brain template and atlas, specifically designed for brains that were pre-processed and tissue cleared for 3D optical imaging. To overcome differential volumetric and morphological effects related to tissue clearing, two versions of the template were created: OCUM for BABB/Murray’s clear and iOCUM for DBE/DISCO protocols. The utility and application of both templates were then illustrated by detailed anatomical brain mapping of several distinct 3D optical signals.

To optimally design OCUM and iOCUM as MR-based template atlases, we used the DSURQE template and atlas (Dorr et al., 2008; Richards et al., 2011; Ullmann et al., 2013; Steadman et al., 2014; Qiu et al., 2018) as a starting point. Although the AIBS CCFv3 has often been used as a reference for brain atlases in several publications involving LSFM (Salinas et al., 2018; Perens et al., 2021), we specifically chose to work with an MR-based template as a starting point, rather than an STPT-based template. Since STPT and optical clearing methods each induce a specific and differential set of deformations and artefacts as compared to the *in vivo* situation (Supplementary Figure S3), this strategy increased the accuracy of brain mapping for our purpose. Furthermore, this way, optical-MR fusion images can be compared with preclinical PET-MR images for the same marker and provide complementary information at higher resolution. Following this approach we developed the following resources: (1) a T1-weighted template image (40 μm^3^ isotropic resolution) defining (i)OCUM space; (2) their associated tissue priors, or TPMs, required to segment and normalize optical images to the template; (3) a whole brain atlas, delineating 336 VOIs; and (4) a detailed protocol of how to employ these resources to create fusion images and identify specific areas containing optical signal using the atlas. Interestingly, the whole workflow employs NifTI files in SPM, a well-known file format and brain analysis software in the neuroscientific community. To our knowledge, this is the first time SPM is being used for the analysis of optical 3D images. In combination with our resources, this will allow for ROI- and voxel-based quantitative analysis of large optical image datasets, both being well-characterized quantification methods for PET and MR. It should be noted that each template is comprised of brain images acquired from both C57Bl/6 J WT and transgenic adult mice, however, all transgenic models were bred on a C57Bl/6 background. The knockout mice used to create the templates did not have any differences in brain size or morphology to C57Bl/6 J WT mice, and therefore did not influence the anatomical precision of either template. Since (i)OCUM is based on normal adult C57Bl/6 mouse brains, its application might not be justified when using mice with severe brain defects or altered brain morphology and optical data obtained from other mouse strains must be cautiously handled.

The fact that different tissue clearing methods exert differential effects on brain size and morphology has become a generally accepted concept (Kim et al., 2018; Wan et al., 2018). In this study, we specifically chose to work with BABB/Murray’s clear and DBE/iDISCO, two solvent-based clearing methods, over aqueous-based clearing methods such as CUBIC and CLARITY. Solvent-based clearing methods reach the highest level of tissue transparency and shrink the tissue which is beneficial for imaging large tissues such as the rodent brain (Erturk et al., 2012; Qi et al., 2019). The percentage of transmittance is significantly lower for light of all wavelengths using CUBIC compared to Murray’s clear and iDISCO (Qi et al., 2019). In addition, CLARITY does not allow for antibody staining prior to clearing. Our head-to-head comparison showed significant volume differences between BABB and DBE-cleared brains (Figure 2), while no significant differences in brain volume were detected when calculated using tissue autofluorescence (Supplementary Figure S1). Interestingly, there was no significant difference in DBE cleared brain volume calculated from autofluorescence or T1-weighted images, while for BABB cleared brains, volumes calculated from T1-weighted images were even slightly larger than *in vivo* brains. These data suggest a differential effect of clearing agents on tissue rehydration rather than on shrinkage. Another factor we identified to impact brain size of optical images, potentially rendering the co-registration/normalization workflow more challenging, was the zoom factor used during whole brain optical imaging. With our OPT setup, we repeatedly observed near perfect fusion results on images acquired with an optical zoom factor of 1.25 and 1.6 during image acquisition, while images acquired with larger zoom factors were less accurate after normalization, likely due to additional skewing to OCUM. In line with previous reports mapping optical 3D signal to brain templates (Perens et al., 2021), we experienced more difficulty in automatically delineating the cortical ROIs located in the hind brain and had to adapt them manually to fit, while this procedure was not required in anterior cortical nor subcortical regions, implying differential effects of clearing agents throughout the brain.

The creation of optical-MR fusion images, implies bringing the optical signal to the MR template reference space, adapting both the template’s bounding box and voxel dimension. This has several implications for optical images. While OCUM is a high-resolution template (40 μm^3^), the original OPT-images in this study have a voxel size ranging from 16.5–21 μm^3^. This means that the voxel size of optical images is increased, thus lowering their resolution to create fusion images. LSFM has higher resolution compared to OPT, resulting in a greater loss of resolution when fit into the template space. Furthermore, while OPT generates isotropic voxels (identical dimension in x, y, and z), LSFM has lower axial than lateral resolution, resulting in anisotropic voxels. LSFM images can be first resampled along the z-axis to reach isotropic voxel size and then co-registered and normalized to the anatomical template. Nevertheless, this may lead to ambiguities in axial direction due to signal skewing during resampling, which may greatly impact voxel-wise quantitative analyses but may even exert a clear effect on the ROI level. However, great advances are being made both for software and hardware in this field. In 2019, Chakraborty et al. described cleared-tissue axially swept light-sheet microscopy (ctASLM) wherein z-axial resolution is significantly increased which results in approximate isotropic voxels (Chakraborty et al., 2019).

To exemplify the possibilities of our resources, we created fusion images of OPT and LSFM images with OCUM and iOCUM. Using both templates, we created fusion images of DAT expression and identified signal in the striatum, amygdala, olfactory tubercle, hypothalamus and substantia nigra. All these regions are known DAT expressing regions, which clearly highlights the applicability of our resources. Although beyond the scope of this study, we only observed DAT signal on the outermost surface of striatum, likely due to antibody competition induced by the high striatal expression levels of dopamine transporter. Furthermore, we identified specific brain regions that were susceptible for flaviviral infection both using OCUM and iOCUM. Our results were in line with a previous report where viral distribution patterns were visualized and quantified using our approach (Chotiwan et al., 2023). Using OCUM, we also traced anterograde innervation from the eye along the optic tract and observed a match to brain regions from the visual system. Finally, we visualized TSPO expression by OPT and LSFM and observed high expression in the 4th cortical layer, indicating high levels of microglial activation, and extremely high signal in the vessels. Although it is known that TSPO is also expressed in endothelial cells (Guilarte et al., 2022) TSPO PET does not show this feature due to its limited resolution while OPT and LSFM showed the extent of vessel binding of this important neuroinflammation marker. These examples highlight the potential of this technique to discover novel biological insights among different brain systems and brain diseases. Together, this demonstrates how our resources can aid spatial and quantitative analyses of treated versus control animals or for a cross-sectional quantitation of specific disease markers over time. Finally, it may serve in the further development of machine-learning approaches for optical imaging (Todorov et al., 2020).

## Data availability statement

The raw data supporting the conclusions of this article will be made available by the authors, without undue reservation.

## Ethics statement

The animal studies were either approved by the regional Animal Research Ethics Committee of Northern Norrland, or by the Animal Review Board at the Court of Appeal of Northern Stockholm and the Swedish Board of Agriculture. The study was conducted in accordance with the local legislation and institutional requirements.

## Author contributions

SW: Writing – review & editing, Writing – original draft, Visualization, Validation, Software, Resources, Project administration, Methodology, Investigation, Formal analysis, Data curation, Conceptualization. FM: Writing – review & editing, Methodology, Investigation, Data curation. TM: Writing – review & editing, Methodology, Investigation, Formal analysis, Data curation. EN: Writing – review & editing, Investigation, Data curation. GO: Writing – review & editing, Methodology, Data curation. MH: Writing – review & editing, Data curation. NC: Writing – review & editing, Data curation. MV: Writing – review & editing, Methodology, Data curation. P-OB: Writing – review & editing, Supervision, Resources, Funding acquisition. EI: Writing – review & editing, Supervision, Data curation. AÖ: Writing – review & editing, Supervision, Funding acquisition. UA: Writing – review & editing, Visualization, Supervision, Software, Resources, Methodology, Funding acquisition. DM: Writing – review & editing, Writing – original draft, Visualization, Validation, Supervision, Software, Resources, Project administration, Methodology, Investigation, Funding acquisition, Formal analysis, Data curation, Conceptualization.
