# Supplementary material for: An MR-based brain template and atlas for optical projection tomography and light sheet fluorescence microscopy in neuroscience
Source: Front Neurosci. 2024 Mar 27;18:1328815. doi: 10.3389/fnins.2024.1328815 (PMC11004350; doi:10.3389/fnins.2024.1328815)
Supplement: Supplementary file 1 [file Data_Sheet_1.DOCX]

Supplementary Material

An MR-based brain template and atlas for Optical Projection Tomography and Light sheet Fluorescence Microscopy in neuroscience

Stefanie Willekens^1,2,3*^, Federico Morini^3^, Tomas Mediavilla^4^, Emma Nilsson^1,2^, Greger Orädd^4^, Max Hahn^3^, Nunya Chotiwan^1,2^, Montse Visa^5^, Per-Olof Berggren^5^, Erwin Ilegemns^5^, Anna K. Överby^1,2^, Ulf Ahlgren^3^ and Daniel Marcellino^4*^

^1^Department of Clinical Microbiology, Umeå University, Umeå, Sweden

^2^The Laboratory for Molecular Infection Medicine Sweden (MIMS), Umeå University, Umeå, Sweden

^3^Umeå Centre for Molecular Medicine, Umeå University, Umeå, Sweden

^4^Department of Integrative Medical Biology, Umeå University, Umeå, Sweden

^5^The Rolf Luft Research Center for Diabetes and Endocrinology, Karolinska Institutet, Stockholm, Sweden

*** Correspondence:** Stefanie Willekens: stefanie.willekens@umu.se

Daniel Marcellino: daniel.marcellino@umu.se

# Supplementary Data

No supplementary Data

# Supplementary Figures and Tables

## Supplementary Figures


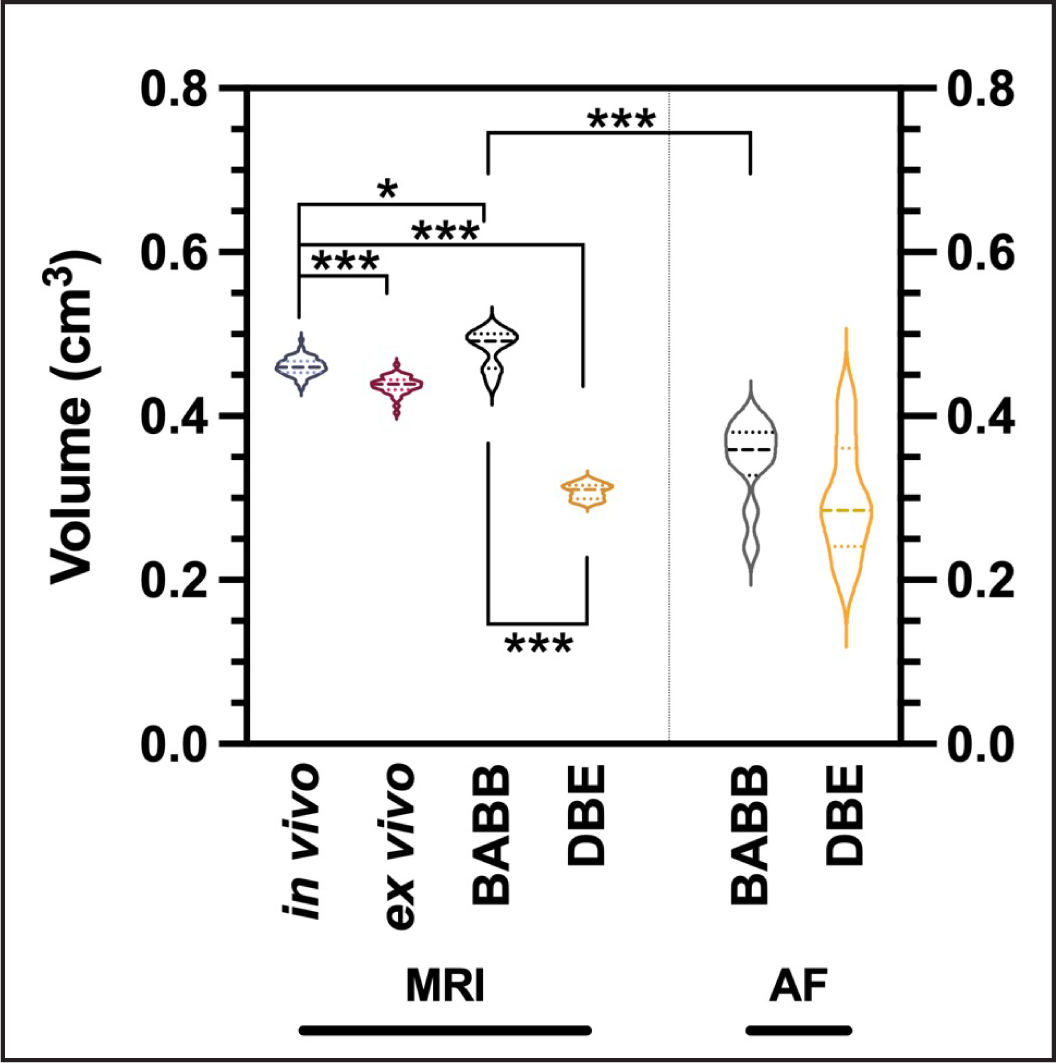


**Supplementary figure 1: Differential effects of clearing methods and rehydration on brain volume.** Brain volume calculations of the average BABB and DBE brains with average *in vivo* and *ex vivo in situ* brain volumes, as well as with their respective autofluorescence volumes. All values are expressed in cm^3^. The average brain volume was significantly lower (***p<0.001) for DBE cleared brains (0.308 ± 0.009 cm^3^) as compared to BABB cleared brains (0.483 ± 0.023 cm^3^). The *in vivo* brain volume showed significant differences (***p<0.001; *p=0.03) with all other calculated brain volumes. Comparison of the average BABB and DBE brain sizes, based on autofluorescence, indicated no difference in brain size after optical clearing. Comparison of these volumes with their respective volumes after rehydration for MR-acquisition, indicated that the BABB brain volume was significantly larger when calculated based on T1-weighted images (0.483 ± 0.023 cm^3^) as when calculated from tissue (p<0.001) autofluorescence (0.343 ± 0.0517 cm^3^), which was not the case for the DBE cleared brains (p=0.94).

**
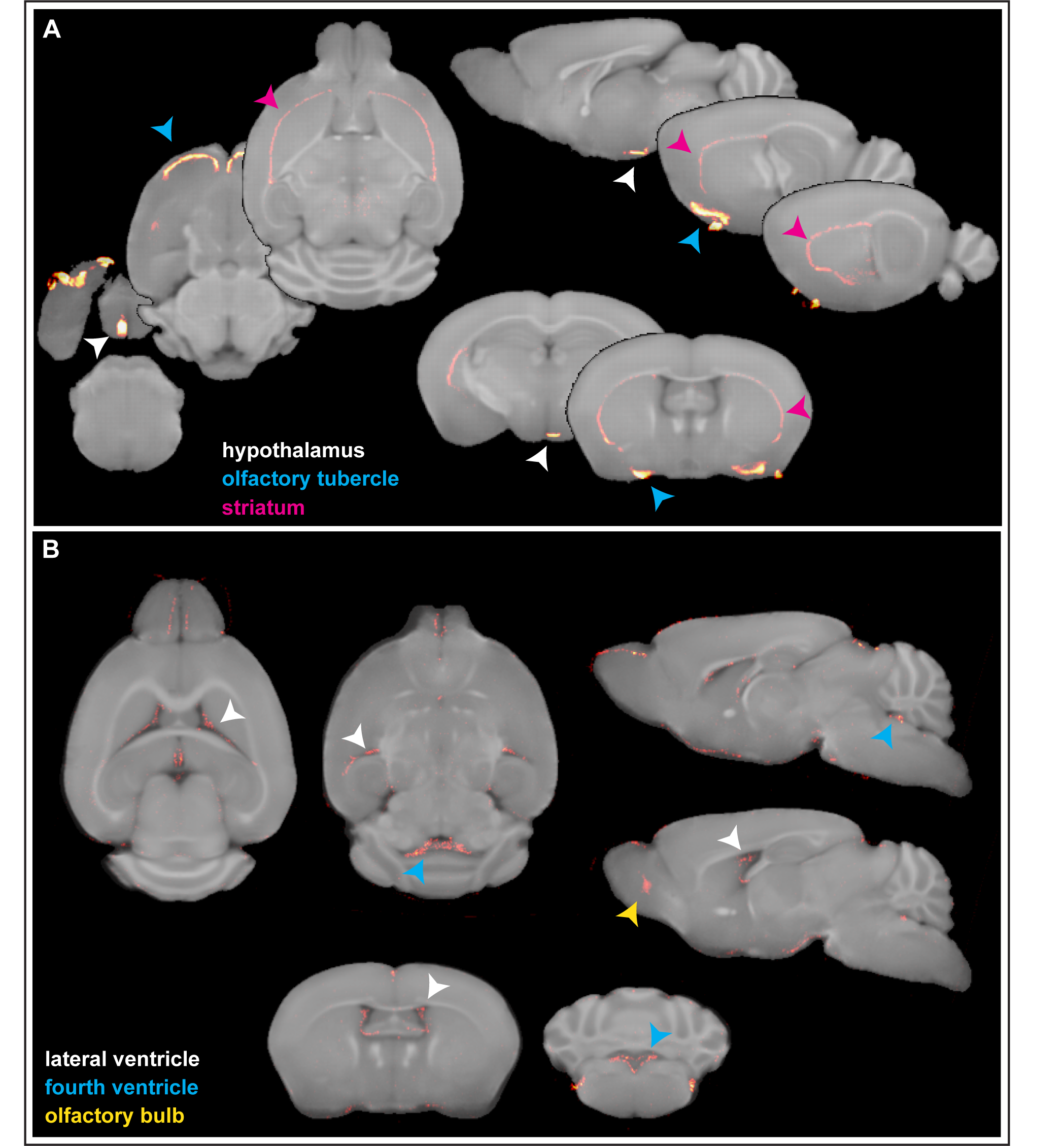
**

**Supplementary figure 2: Fusion images of 3D optical DAT and viral signal with the iOCUM template.** A) 2D representations of fusion images of DAT OPT signal in typical dopamine transporter expressing brain regions and iOCUM. B) 2D and 3D representations of fusion images of viral OPT signal in infected brain regions and iOCUM.

**
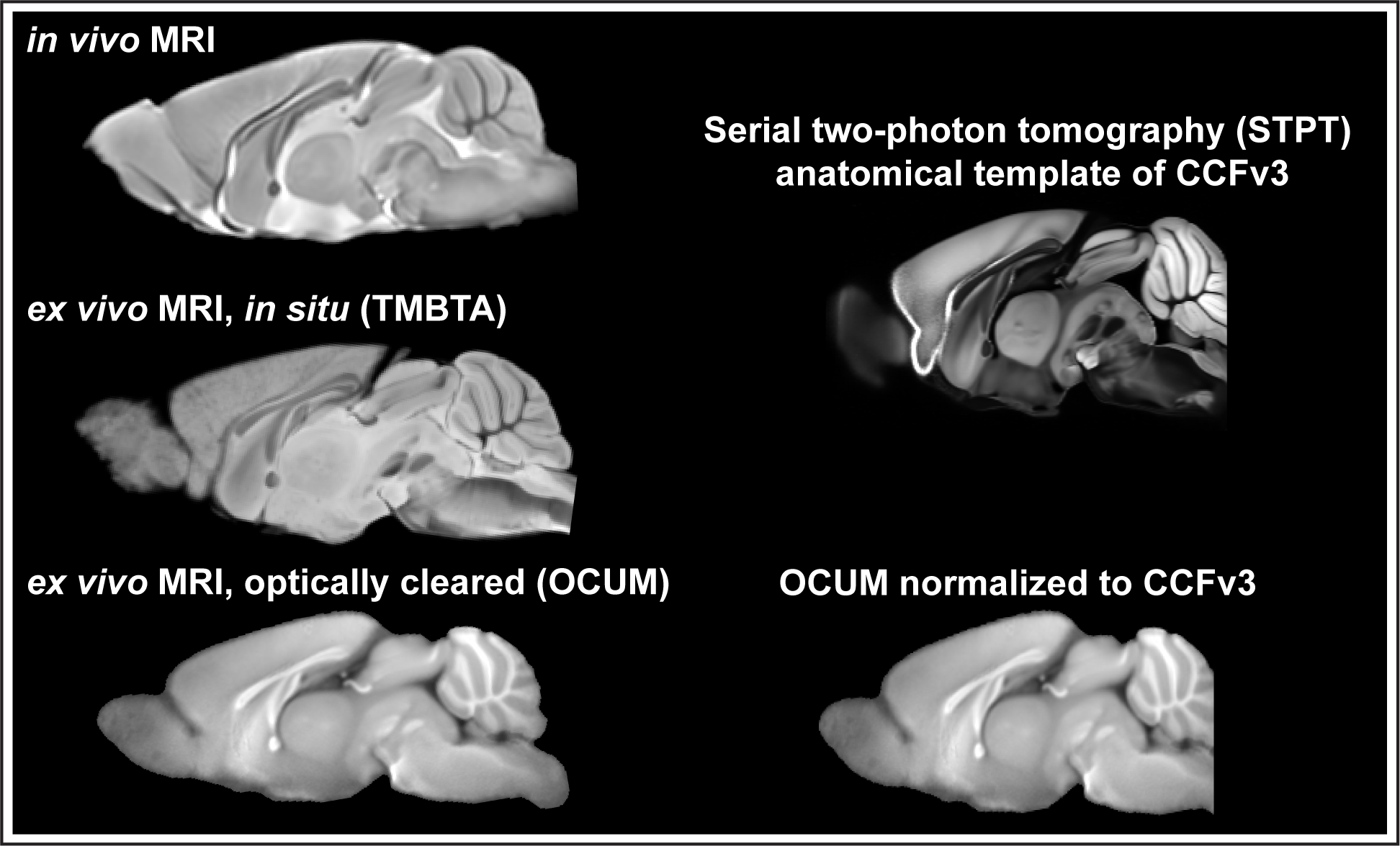
**

**Supplementary figure 3: Comparison of brain morphology among anatomical brain templates.** Different deformations are caused by different imaging modalities (MRI and STPT) and tissue processing (*ex vivo* *in situ* and *ex vivo* cleared), as compared to *in vivo* native brain morphology.

## Supplementary Tables

**Supplementary Table 1:** OCUM and iOCUM atlas labels

|  | Structure | Right Label | Left Label |
| --- | --- | --- | --- |
| 1 | Amydala | 51 | 151 |
| 2 | Anterior commissure: Olfactory Limb | 115 | 215 |
| 3 | Anterior Commissure: Temporal Limb | 23 | 103 |
| 4 | Ventral Pallidum (basal forebrain) | 52 | 152 |
| 5 | Bed nucleus of stria Terminalis | 176 | 76 |
| 6 | Inferior cerebellar peduncle | 123 | 223 |
| 7 | Middle cerebellar peduncle | 45 | 245 |
| 8 | Superior cerebellar peduncle | 242 | 222 |
| 9 | Cerebral aqueduct | 119 | 119 |
| 10 | Cerebral peduncle | 114 | 14 |
| 11 | Inferior colliculus | 143 | 43 |
| 12 | Superior colliculus | 9 | 109 |
| 13 | Corpus callosum | 8 | 68 |
| 14 | Corticospinal tract | 218 | 18 |
| 15 | Cuneate nucleus | 166 | 168 |
| 16 | Facial nerve | 19 | 219 |
| 17 | Fasciculus retroflexus | 25 | 125 |
| 18 | Fimbria | 211 | 11 |
| 19 | Fornix | 122 | 22 |
| 20 | Fourth ventricle | 118 | 118 |
| 21 | Fundus of striatum | 54 | 154 |
| 22 | Dorsal pallidum (globus pallidus) | 44 | 144 |
| 23 | Habenular commissure | 99 | 199 |
| 24 | Hypothalamus | 250 | 150 |
| 25 | Inferior olivary complex | 113 | 2013 |
| 26 | Internal capsule | 112 | 12 |
| 27 | Interpeduncular nucleus | 157 | 157 |
| 28 | Lateral olfactory tract | 101 | 102 |
| 29 | Lateral septum | 207 | 207 |
| 30 | Lateral ventricle | 57 | 77 |
| 31 | mammillary bodies | 161 | 61 |
| 32 | mammilothalamic tract | 210 | 212 |
| 33 | Medial Lemniscus | 20 | 120 |
| 34 | Medial septum | 53 | 153 |
| 35 | Medulla | 174 | 174 |
| 36 | Midbrain | 194 | 194 |
| 37 | Nucleus Accumbens | 55 | 155 |
| 38 | Olfactory peduncle | 5 | 105 |
| 39 | Olfactory tubercle | 95 | 145 |
| 40 | Optic tract | 216 | 116 |
| 41 | Periaqueductal grey | 10 | 10 |
| 42 | Pons | 187 | 187 |
| 43 | Pontine nucleus | 85 | 185 |
| 44 | Posterior commissure | 100 | 100 |
| 45 | Subiculum | 133 | 131 |
| 46 | Stria medullaris | 225 | 205 |
| 47 | Stria terminalis | 59 | 159 |
| 48 | Striatum | 7 | 17 |
| 49 | Subpendymale zone | 240 | 140 |
| 50 | Superior olivary complex | 124 | 214 |
| 51 | Thalamus | 204 | 4 |
| 52 | Third ventricle | 146 | 146 |
| 53 | Ventral tegmental decussation | 156 | 156 |
| 54 | Cerebellar vermis lobules 1-2 Lingula and ventral central | 32 | 32 |
| 55 | Cerebellar vermis lobule 3: Dorsal central | 233 | 233 |
| 56 | Cerebellar vermis lobules 4-5: culmen | 34 | 34 |
| 57 | Cerebellar vermis lobule 6: declive | 36 | 36 |
| 58 | Cerebellar vermis lobule 7: tuber/folium | 237 | 237 |
| 59 | Cerebellar vermis lobule 8: pyramus | 38 | 38 |
| 60 | Cerebellar vermis lobule 9: uvula | 239 | 239 |
| 61 | Cerebellar vermis lobule 10: nodulus | 40 | 40 |
| 62 | Cerebellar paravermis lobules 4-5: anterior lobule | 90 | 148 |
| 63 | Cerebellar hemisphere lobule 6: simple lobule | 191 | 91 |
| 64 | Cerebellar hemisphere lobule 6: ansiform lobule (crus 1) | 92 | 192 |
| 65 | Cerebellar hemisphere lobule 7: ansiform lobule (crus 2) | 193 | 93 |
| 66 | Cerebellar hemisphere lobule 7: paramedian lobule | 94 | 200 |
| 67 | Cerebellar hemisphere lobule 8: copula pyramidis | 196 | 96 |
| 68 | Flocculus | 97 | 197 |
| 69 | Paraflocculus | 198 | 98 |
| 70 | Trunk of arbor vita | 47 | 47 |
| 71 | Cerebellar vermis WM: lobules 1-2 | 232 | 232 |
| 72 | Cerebellar vermis WM: lobule 3 | 33 | 33 |
| 73 | Cerebellar vermis WM: trunk of lobules 1-3 | 253 | 253 |
| 74 | Cerebellar vermis WM: lobules 4-5 | 234 | 234 |
| 75 | Cerebellar vermis WM: lobules 6-7 | 236 | 236 |
| 76 | Cerebellar vermis WM: lobule 8 | 238 | 238 |
| 77 | Cerebellar vermis WM: trunk of lobules 6-8 | 254 | 254 |
| 78 | Cerebellar vermis WM: lobule 9 | 139 | 139 |
| 79 | Cerebellar vermis WM: lobule 10 | 252 | 252 |
| 80 | Cerebellar paravermis WM: anterior lobule | 21 | 31 |
| 81 | Cerebellar WM: simple lobule | 241 | 251 |
| 82 | Cerebellar WM: crus 1 | 220 | 170 |
| 83 | Cerebellar WM: trunk of simple and crus 1 | 226 | 246 |
| 84 | Cerebellar WM: crus 2 | 229 | 249 |
| 85 | Cerebellar WM: paramedian lobule | 228 | 248 |
| 86 | Cerebellar WM: trunk of crus 2 and paramedian | 175 | 195 |
| 87 | Cerebellar WM: copula | 224 | 244 |
| 88 | Paraflocculus WM | 183 | 243 |
| 89 | Flocculus WM | 167 | 177 |
| 90 | Dentate nucleus | 1 | 201 |
| 91 | Nucleus interpositus | 203 | 3 |
| 92 | Fastigial nucleus | 15 | 206 |
| 93 | Cingulate cortex: area 24a | 24 | 169 |
| 94 | Cingulate cortex: area 24a' | 26 | 171 |
| 95 | Cingulate cortex: area 24b | 27 | 172 |
| 96 | Cingulate cortex: area 24b' | 28 | 173 |
| 97 | Cingulate cortex: area 25 | 29 | 178 |
| 98 | Cingulate cortex: area 29a | 30 | 179 |
| 99 | Cingulate cortex: area 29b | 35 | 182 |
| 100 | Cingulate cortex: area 29c | 37 | 184 |
| 101 | Cingulate cortex: area 30 | 39 | 186 |
| 102 | Cingulate cortex: area 32 | 41 | 188 |
| 103 | Amygdalopiriform transition area | 42 | 189 |
| 104 | Primary auditory cortex | 46 | 208 |
| 105 | Dorsal Secondary auditory cortex | 48 | 217 |
| 106 | Ventral Secondary auditory cortex | 49 | 221 |
| 107 | Caudomedial entorhinal cortex | 50 | 227 |
| 108 | Cingulum | 56 | 231 |
| 109 | Claustrum | 58 | 235 |
| 110 | Piriform area | 60 | 255 |
| 111 | Dorsal Claustrum | 62 | 256 |
| 112 | Dorsal Endopiriform nucleus | 65 | 257 |
| 113 | Dorsal intermediate entorhinal cortex | 67 | 258 |
| 114 | Dorsolateral entorhinal cortex | 69 | 259 |
| 115 | Dorsolateral orbital cortex | 70 | 260 |
| 116 | Dorsal tenia tecta | 71 | 261 |
| 117 | Ectorhinal cortex | 72 | 262 |
| 118 | Frontal cortex: area 3 | 73 | 263 |
| 119 | Frontal association cortex | 74 | 264 |
| 120 | Intermediate nucleus of endopiriform claustrum | 75 | 265 |
| 121 | Insular region: not subdivided | 78 | 266 |
| 122 | Lateral orbital cortex | 79 | 267 |
| 123 | Lateral parietal association cortex | 80 | 268 |
| 124 | Primary motor cortex | 81 | 269 |
| 125 | Secondary motor cortex | 82 | 270 |
| 126 | Medial entorhinal cortex | 83 | 271 |
| 127 | Medial orbital cortex | 84 | 272 |
| 128 | Medial parietal association cortex | 86 | 273 |
| 129 | Piriform cortex | 87 | 274 |
| 130 | Posterolateral cortical amygdaloid area | 88 | 275 |
| 131 | Posteromedial cortical amygdaloid area | 89 | 176 |
| 132 | Perirhinal cortex | 104 | 277 |
| 133 | arietal cortex: posterior area: rostral part | 108 | 278 |
| 134 | Rostral amygdalopiriform area | 110 | 279 |
| 135 | Primary somatosensory cortex | 111 | 280 |
| 136 | Primary somatosensory cortex: barrel field | 117 | 281 |
| 137 | Primary somatosensory cortex: dysgranular zone | 121 | 282 |
| 138 | Primary somatosensory cortex: forelimb region | 126 | 283 |
| 139 | Primary somatosensory cortex: hindlimb region | 127 | 284 |
| 140 | Primary somatosensory cortex: jaw region | 128 | 285 |
| 141 | Primary somatosensory cortex: shoulder region | 129 | 286 |
| 142 | Primary somatosensory cortex: trunk region | 132 | 287 |
| 143 | Primary somatosensory cortex: upper lip region | 134 | 288 |
| 144 | Secondary somatosensory cortex | 135 | 289 |
| 145 | Temporal association area | 136 | 290 |
| 146 | Primary visual cortex | 137 | 291 |
| 147 | Primary visual cortex: binocular area | 138 | 292 |
| 148 | Primary visual cortex: monocular area | 141 | 293 |
| 149 | Secondary visual cortex: lateral area | 142 | 294 |
| 150 | Secondary visual cortex: mediolateral area | 147 | 295 |
| 151 | Secondary visual cortex: mediomedial area | 149 | 296 |
| 152 | Ventral Claustrum | 158 | 297 |
| 153 | Ventral nucleus of the endopiriform claustrum | 160 | 298 |
| 154 | Ventral intermediate entorhinal cortex | 162 | 299 |
| 155 | Ventral orbital cortex | 163 | 300 |
| 156 | Ventral tenia tecta | 165 | 301 |
| 157 | Hippocampal region: CA10r | 336 | 305 |
| 158 | Hippocampal region: LMol | 306 | 307 |
| 159 | Hippocampal region: CA1Rad | 308 | 309 |
| 160 | Hippocampal region: CA2Py | 310 | 311 |
| 161 | Hippocampal region: CA20r | 312 | 313 |
| 162 | Hippocampal region: CA2Rad | 314 | 315 |
| 163 | Hippocampal region: CA3Py Inner | 316 | 317 |
| 164 | Hippocampal region: CA3Py Outer | 318 | 319 |
| 165 | Hippocampal region: CA30r | 320 | 321 |
| 166 | Hippocampal region: CA3Rad | 322 | 323 |
| 167 | Hippocampal region: SLu | 324 | 325 |
| 168 | Hippocampal region: MoDG | 326 | 327 |
| 169 | Hippocampal region: GrDG | 328 | 329 |
| 170 | Hippocampal region: PoDG | 330 | 331 |
| 171 | Hippocampal region: CA1Py | 334 | 335 |
| 172 | Olfactory bulb: glomerular layer | 337 | 345 |
| 173 | Olfactory bulb: external plexiform layer | 338 | 346 |
| 174 | Olfactory bulb: mitral cell layer | 339 | 347 |
| 175 | Olfactory bulb: internal plexiform layer | 340 | 348 |
| 176 | Olfactory bulb: granule cell layer | 341 | 349 |
| 177 | Accessory olfactory bulb: glomerular, external plexiform and mitral cell layer | 342 | 350 |
| 178 | Accessory olfactory bulb: granule cell layer | 343 | 351 |
| 179 | Anterior olfactory nucleus | 344 | 352 |
| 180 | subiculum | 332 | 333 |
| 181 | Medial amygdala | 353 | 355 |
| 182 | Medial preoptic nucleus | 354 | 356 |
